# Impact of Fast-Acting Insulin Aspart on Glycemic Control in Patients with Type 1 Diabetes Using Intermittent-Scanning Continuous Glucose Monitoring Within a Real-World Setting: The GoBolus Study

**DOI:** 10.1089/dia.2020.0360

**Published:** 2021-02-25

**Authors:** Thomas Danne, Matthias Axel Schweitzer, Winfried Keuthage, Stefan Kipper, Yasmin Kretzschmar, Jörg Simon, Tanja Wiedenmann, Ralph Ziegler

**Affiliations:** ^1^ Diabetes Center for Children and Adolescents, Children's Hospital on the Bult, Hanover Medical School, Hanover, Germany.; ^2^ Novo Nordisk Pharma GmbH, Mainz, Germany.; ^3^ Schwerpunktpraxis für Diabetes und Ernährungsmedizin, Muenster, Germany.; ^4^ Medizinisches Versorgungszentrum im Altstadt-Carree Fulda GmbH, Fulda, Germany.; ^5^ Diabetes Clinic for Children and Adolescents, Muenster, Germany.

**Keywords:** Diabetes mellitus, Continuous glucose monitoring, Prandial insulin, Time-in-range

## Abstract

***Background:*** The GoBolus study investigated the real-world effectiveness of faster aspart in patients with type 1 diabetes (T1D) using intermittent-scanning continuous glucose monitoring (iscCGM) systems.

***Methods:*** This 24-week, multicenter, single-arm, noninterventional study investigated adults with T1D (HbA_1c_, 7.5%–9.5%) receiving multiple daily injections (MDI) of insulin and using iscCGM within local healthcare settings for ≥6 months before switching to faster aspart at study start (week 0; baseline). Primary endpoint was HbA_1c_ change from baseline to week 24. Exploratory endpoint was change in iscCGM metrics from baseline to week 24.

***Results:*** Overall, 243 patients were included (55.6% male), with mean age/diabetes duration, 49.9/18.8 years; mean HbA_1c_, 8.1%. By week 24, HbA_1c_ had decreased by 0.19% (−2.1 mmol/mol, *P* < 0.0001) with no mean change in insulin doses or basal/bolus insulin ratios. For patients with sufficient available iscCGM data (*n* = 92): “time in range” (TIR; 3.9–10.0 mmol/L) increased from 46.9% to 50.1% (*P* = 0.01), corresponding to an increase of 46.1 min/day; time in hyperglycemia decreased from 49.1% to 46.1% (>10.0 mmol/L, *P* = 0.026) and 20.4% to 17.9% (>13.9 mmol/L, *P* = 0.013), corresponding to 43.5 (*P* = 0.024) and 35.6 (*P* = 0.015) fewer minutes per day on average spent in these ranges, respectively; no change for time in hypoglycemia (<3.9 and <3.0 mmol/L). Mean interstitial and postprandial glucose improved from 10.4 to 10.1 mmol/L (*P* = 0.035) and 11.9 to 11.0 mmol/L (*P* = 0.002), respectively.

***Conclusion:*** Real-world switching to faster aspart in adults with T1D on MDI improved HbA_1c_, increased TIR, and decreased time in hyperglycemia without affecting time in hypoglycemia.

The GoBolus study: NCT03450863.

## Introduction

Type 1 diabetes (T1D) is a chronic disease, characterized by absent or near absent beta-cell function, that requires insulin therapy.^1^ Patients with T1D are often treated with a regimen of multiple daily injections (MDI), comprising a long-acting basal component and a mealtime bolus component of short-acting insulin.^1^ The development of faster-acting insulin analogs for prandial dosing increases the treatment options for patients, potentially offering improved glycemic control and more flexibility with dose timing.^1–5^

Fast acting insulin aspart (faster aspart) is an enhanced formulation of insulin aspart (IAsp) containing the excipients niacinamide and l-arginine.^6^ Niacinamide increases the initial abundance of IAsp monomers after subcutaneous administration and mediates a transient, local vasodilatory effect.^6^ A post hoc analysis of pooled data from six pharmacology trials in adults with T1D showed that faster aspart had an ∼5-min earlier onset of appearance in the circulation, a twofold greater early insulin exposure, and a 74% greater early glucose-lowering effect in the first 30 min after injection, compared with IAsp.^7^ The efficacy and safety of faster aspart was investigated in the “onset” clinical trial program and, as part of this, its clinical utility in adult patients receiving MDI was established.^2,4,8–10^ Faster aspart was demonstrated to be noninferior to IAsp with respect to reductions in HbA_1c_.^2,8,11^ Adult and pediatric patients with T1D and adults with type 2 diabetes who were treated with faster aspart experienced improved postprandial glucose (PPG) control and similar or lower rates of hypoglycemia compared with IAsp.^2,8,11^ Moreover, the magnitude of improvements in PPG control with faster aspart versus IAsp are similar to those observed when earlier trials compared short-acting insulin analogs with regular human insulin.^12,13^ Faster aspart therefore represents an important addition to the available treatment options for T1D. Other ultrafast acting insulins are also in development.^14^

The “onset” clinical trials used a combination of self-measured blood glucose monitoring and continuous glucose monitoring (CGM) to investigate the efficacy of faster aspart,^2,3,5,8–11^ with the latter, newer method being particularly useful to examine PPG control. CGM yields real-time information that allows identification of acute glycemic excursions, and analysis of intra- and interday glucose variability and time in range (TIR).^15^ Hence, these data are a valuable addition to traditional HbA_1c_ measurements. “Intermittent-scanning continuous glucose monitoring” (iscCGM) systems (also known as flash glucose monitoring) are becoming widely used as a self-management tool for patients, since they are easy to use and do not require finger-prick calibration.^16,17^ All together, these systems provide comprehensive, meaningful data that allow patients and healthcare professionals to react faster to glucose perturbations or trends and better enable them to closely monitor the outcomes of treatment adjustments or switches.

Most of the clinical data on faster aspart are from the controlled “onset” treat-to-target clinical trials; there is limited real-world evidence of the impact of faster aspart. The GoBolus study, which is the first real-world evidence study investigating faster aspart, was therefore designed to analyze the real-world effectiveness and safety of faster aspart in patients with T1D on MDI who were using iscCGM, and thus examine if the observations of the “onset” trials translate to clinical practice. An exploratory analysis also investigated the change in iscCGM metrics from baseline.

## Materials and Methods

### Study design

GoBolus (NCT03450863) was a 24-week, multicenter, single-arm, observational study conducted in Germany with retrospective data extraction and prospective data collection to assess the effectiveness of faster aspart in adult patients with T1D using iscCGM. The study was noninterventional, as prescription of faster aspart was independent of this study and at the discretion of the treating physician as part of their usual clinical practice. Diagnostic or monitoring procedures outside of usual clinical practice were not applied.

The study included three visits in line with the local clinical practice: an initiation visit, which was the starting point of treatment with faster aspart (visit 1; week 0; baseline), a mid-study visit (visit 2; 12 ± 4 weeks), and an end-of-study visit (visit 3; 24 ± 4 weeks). The study recruited patients who were to be started on faster aspart as part of their usual clinical practice, and it was initiated on March 22, 2018 (first patient, first visit) and completed on September 4, 2019 (last patient, last visit).

The study was conducted in accordance with International Conference on Harmonization Good Clinical Practice Guidelines^18^ and the Declaration of Helsinki.^19^ Informed consent was obtained before any trial-related activities. Patients received complete information about the study both verbally and in writing. The study received a professional legal consultation according to §15 of the professional regulations for doctors. The primary consultation was done by the Ethics Committee of the state medical association of Bavaria with the registration number 17089 on January 25, 2018. Further respective approvals were received by other relevant regional Ethics Committees.

### Participants

The inclusion and exclusion criteria for study participation were sufficiently broad to help generalizability of study results to the wider adult population of patients with T1D using faster aspart treatment in local healthcare settings in Germany.

Patients included were adults (≥18 years old), diagnosed with T1D at least 1 year before study inclusion and on stable insulin treatment with MDI for at least the previous 6 months before inclusion in this study, with HbA_1c_ between 7.5% and 9.5% at the latest reading (in the last 3 months). The upper limit of HbA_1c_ levels was set to 9.5% to exclude patients who needed basal insulin optimization, as the study aimed to observe the impact of the change in bolus insulin treatment. Patients were also required to be regular users (defined as usage on a monthly basis) of iscCGM for at least 6 months before study inclusion (visit 1; week 0; baseline).

Key exclusion criteria included antidiabetic treatment intensification (defined as adding new antidiabetic medication to previous treatment regimen) during the 3 months before study start, and women who were pregnant, breast feeding, or where pregnancy during the study was a possibility.

The analysis sets were defined as follows: the full analysis set (FAS) included all enrolled patients, defined as all those who had signed an informed consent for the study, excluding screening failures; the safety analysis set (SAS) included all patients who had received at least one dose of study treatment; the effectiveness analysis set (EAS) included all patients from the SAS without relevant protocol deviations and who continued on treatment until visit 3 of the study; and for the primary endpoint EAS (EAS-P), the visit window for visit 3 was extended in a stepwise manner on a weekly basis in line with the statistical analysis plan, until the required group size for the primary endpoint was reached after 24 ± 7 weeks. Protocol deviations were as follows: the latest HbA_1c_ value was not measured in the 3 months before study inclusion (week 0; visit 1; baseline); or HbA_1c_ assessment was after treatment initiation with faster aspart; or HbA_1c_ value at baseline was <7.5% or >9.5%; or visit 3 not performed at 24 ± 4 weeks.

### Procedures

Patients were treated with commercially available faster aspart as bolus insulin injections (MDI). Dosing with faster aspart was individual and determined by the treating physician in accordance with the needs of the patient. All other antidiabetic medications were prescribed at the discretion of the treating physician under routine clinical practice conditions.

The iscCGM monitoring device used in this study was the Freestyle Libre^®^ (Abbott, IL, USA). A period of at least 14 days of retrospective data collection (a full 2 weeks sensor download as csv file with at least 80% completeness of the data) was required to allow sufficient analysis of isCGM data before each visit. If a data set was found with less than 80% completeness, the previous date of sensor change before the evaluated period was chosen to determine the start of the previous measurement period. To find a period with sufficient completeness, this process was performed up to three times at baseline (visit 1; week 0), then up to two times at weeks 12 (visit 2) and 24 (visit 3). The iscCGM data were checked for the following: to identify systematic gaps (data missing for more than 4.8 h on the same period of 3 or more days) and possible wrong measurements (e.g., values of >22.2 mmol/L [>400 mg/dL] were checked); to verify that times and dates were in the expected format and aligned with visit dates/sensor changes; to verify that iscCGM units were the same for all participants; and to check for extra data and duplicate time stamps.

### Outcome measures

The primary endpoint of this study was change in HbA_1c_ from baseline (week 0) to week 24. In cases where fasting plasma glucose (FPG)/HbA_1c_ was not measured at the initiation visit (visit 1; week 0; baseline), the latest HbA_1c_ and FPG measurement in the health record within the previous 3 months were used as baseline measurements. HbA_1c_ was measured locally at each site's laboratory.

Secondary endpoints included change from baseline (week 0) to week 12 in HbA_1c_; change from baseline to week 12 and to week 24 in laboratory-measured FPG; and change from baseline to week 24 in the following: total daily basal insulin, bolus insulin, and basal/bolus ratio (defined as total daily basal insulin [U] divided by total daily bolus insulin), timing of insulin administration in relationship to the start of the meal (measured in minutes), total treatment satisfaction score in the Diabetes Treatment Satisfaction Questionnaire (DTSQ) and total treatment-related impact in the Treatment Related Impact Measure for Diabetes (TRIM-D) questionnaire.

Exploratory endpoints included reasons for initiating faster aspart, reasons for premature discontinuation of faster aspart, and the change in iscCGM data from baseline (week 0) to week 12 and week 24 for the FAS dataset. Analyses of iscCGM data with the EAS dataset were done as post hoc analyses.

iscCGM data were described by means of consolidated ambulatory glucose profile (as recommended by international consensus^20^) and endpoints included mean interstitial glucose (measured within 24 h, day and night), TIR (referred to as the time spent in the target glucose range of the patient: 3.9–10 mmol/L [70–180 mg/dL]), time spent in hypoglycemia (<3 mmol/L [<54 mg/dL], <3.9 mmol/L [<70 mg/dL]), time spent in hyperglycemia (>10 mmol/L [>180 mg/dL], >13.9 mmol/L [>250 mg/dL]), PPG (defined as the average glucose raise of two consecutive measurements of a value 2 mmol/L [36 mg/dL] higher than fasting preprandial glucose [FPPG] occurring within 3 h of FPPG), FPPG (defined as the first glucose value after night time [after 05:59 am] before first glucose raise occurring before 09:00 am), estimated HbA_1c_, number and duration of hypoglycemic episodes (glucose alert value <3.9 mmol/L [<70 mg/dL], and clinically significant hypoglycemia <3 mmol/L [<54 mg/dL]) in 24 h. TIR and time in hypo- and hyperglycemia are expressed as the percentage of measurements that are in each of the given glucose ranges and the average minutes per day spent in the given ranges.^15^ Hypoglycemia was defined as at least two consecutive readings at 15-min intervals, <3 mmol/L (54 mg/dL), with the end of an episode represented by two readings at or higher than this threshold. Glycemic variability was evaluated by analyzing the coefficient of variation of the mean daily glucose and mean amplitude of glucose excursion (MAGE).

At each visit, hospitalizations for diabetic ketoacidosis or severe hypoglycemia, the number of nonserious hypoglycemic episodes, serious adverse reactions (SAR), and fatal events or pregnancies were recorded.

Measurements taken at visit 1 (or within previous 3 months of visit 1 for HbA_1c_ and FPG) are hereafter referred to as baseline measurements.

### Statistical analysis

The primary and secondary endpoints were analyzed using a paired *t*-test to assess the statistical significance of the mean change in each parameter from baseline to week 12 or 24.

TRIM-D^21,22^ includes 28 items that are grouped into 5 domains (treatment burden, daily life, diabetes management, compliance, and psychological health), where items are scored from 1 to 5 (higher scores indicate a better outcome). Total treatment-related impact was computed by adding all items and a transformation such that values ranged from 0 to 100. Total treatment-related impact could only be computed if all of the domains could be scored. DTSQ^23,24^ was a six-item patient-reported outcome (based on treatment satisfaction and perceived frequency of hyper- and hypoglycemia) scored on a scale from 0 to 6 (higher scores indicate a better outcome). If one or two items were not answered, total treatment satisfaction could still be computed. Comparisons in change from baseline in scores were performed with paired *t*-tests similar to that used for the primary analysis.

The iscCGM data consisted of the aggregated analysis of data from the patients. CGM metrics were calculated according to the international consensus on CGM metrics.^20^ All comparisons between visits were done by means of paired *t*-tests. All tests performed were two sided with a 5% level of significance.

Safety data, reasons for treatment initiation/premature discontinuation, and change in timing of insulin administration were summarized descriptively.

The study planned to enroll 220 patients with MDI, with assumption rates of 10% for screening failure and 15% for withdrawal (loss of a patient for any reason before completing the 24 weeks observation period).

## Results

The study was conducted in 41 sites in Germany between March 2018 and September 2019. In total, 244 patients were enrolled and 241 were treated with faster aspart (Supplementary Fig. S1). The three main reasons given for initiating faster aspart were to improve the patient's blood glucose profile, insufficient HbA_1c_ adjustment on patient's current regimen, and improved time flexibility in bolus administration (Supplementary Table S1). The main reasons for premature discontinuation are shown in Supplementary Table S2 and include loss to follow-up, which accounted for 4.1% of patients from the SAS.

Baseline characteristics are summarized in Table 1. Of the 155 patients who comprised the EAS, iscCGM data with sufficient data completeness were available for 92 patients (Supplementary Fig. S1; Table 1). Data presented for effectiveness endpoints are from the EAS (EAS-P for primary endpoint, iscCGM-EAS for exploratory endpoints), while safety data are from the SAS (*n* = 241). Data concerning effectiveness endpoints for the FAS can be found in the Supplementary Materials (Supplementary Table S3 and Supplementary Figs. S2–S4).

**Table 1.** Baseline Characteristics of Patients, Full Analysis Set, and Effectiveness Analysis Set

	Total study population		iscCGM subset	
	FAS*^a^*(*N* = 243)	EAS*^b^*(*n* = 155)	iscCGM-FAS*^a^*(*n* = 206)	iscCGM-EAS*^b^*(*n* = 92)
Age, years	49.9 (16.6)	48.9 (16.5)	51.0 (16.7)	52.5 (15.7)
Sex, *n* (%)^c^				
Female	107 (44.0)	77 (49.7)	95 (46.1)	46 (50.0)
Male	135 (55.6)	78 (50.3)	110 (53.4)	46 (50.0)
Body weight, kg	83.5 (18.6)	83.3 (18.7)	82.7 (17.4)	82.5 (16.9)
BMI, kg/m^2^	28.1 (5.6)	28.1 (5.8)	28.0 (5.2)	27.9 (5.1)
Duration of diabetes, years	18.8 (12.4)	18.3 (12.2)	19.2 (12.3)	19.4 (13.2)
HbA_1c_, %	8.1 (0.6)	8.1 (0.6)	8.1 (0.6)	8.1 (0.5)
HbA_1c_ categories, *n* (%)				
<7.5%	9 (3.7)	0	8 (3.9)	0
7.5% to <8.5%	170 (70.0)	117 (75.5)	146 (70.9)	68 (73.9)
8.5% to <9.5%	52 (21.4)	34 (21.9)	44 (21.4)	23 (25.0)
≥9.5%	10 (4.1)	4 (2.6)	7 (3.4)	1 (1.1)
Missing	2 (0.8)	0	1 (0.5)	0
FPG, mg/dL	171.3 (77.5)	166.9 (71.3)	171.6 (75.1)	155.6 (70.4)
FPG, mmol/L^d^	9.5 (4.3)	9.3 (4.0)	9.5 (4.2)	8.6 (3.9)
Prescribed basal insulin dose, U	27.5 (15.0)	27.0 (13.2)	27.2 (14.0)	26.0 (11.2)
Prescribed mealtime insulin dose, U	31.1 (17.4)	31.4 (16.8)	30.7 (16.0)	30.3 (14.9)

Data are mean (±SD) unless otherwise stated.

^a^ FAS: included all enrolled patients, defined as all those who had signed an informed consent for the study, excluding screening failures.

^b^ EAS: included all patients from the SAS without relevant protocol deviations and who continued on treatment until visit 3 of the study.

^c^ One patient had this data missing from the FAS of the iscCGM set.

^d^ Calculated by dividing mg/dL data by 18.02.

BMI, body mass index; EAS, effectiveness analysis set; FAS, full analysis set; FPG, fasting plasma glucose; HbA_1c_, glycated hemoglobin; iscCGM, intermittent-scanning continuous glucose monitoring; iscCGM-EAS, patients with intermittent-scanning continuous glucose monitoring data in the effectiveness analysis set; iscCGM-FAS/EAS, patients with intermittent-scanning continuous glucose monitoring data in the full/effectiveness analysis set; *n*, number of patients; SAS, safety analysis set; SD, standard deviation; U, international unit.

### Primary effectiveness endpoint: change in HbA_1c_ over time

**FIG. 1.**
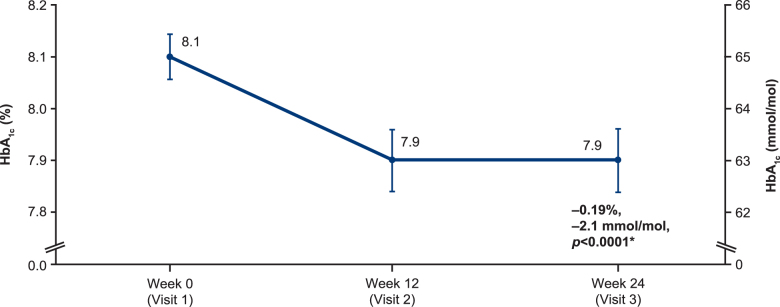
Mean HbA_1c_, EAS-P (*n* = 170). *Mean of the pairwise differences between visits per patient; values depicted are mean ± standard error. EAS-P, extended effectiveness analysis set for the primary endpoint; HbA_1c_, glycated hemoglobin.

Patients receiving faster aspart experienced a significant mean decrease of −0.19% (95% CI: −0.27 to −0.10; *P* < 0.0001) (−2.1 mmol/mol (95% CI: −3.0 to −1.1) in their HbA_1c_ from 8.1% (64.8 mmol/mol) at baseline to 7.9% (62.8 mmol/mol) at week 24 (EAS-P; Fig. 1). The HbA_1c_ at week 12 was also 7.9% (62.8 mmol/mol), which was also a significant reduction from baseline (−0.15; 95% CI: −0.24 to −0.07; *P* = 0.001) (−1.6 mmol/mol (95% CI: −2.6 to −0.8).

### Intermittent-scanning continuous glucose monitoring data

**FIG. 2.**
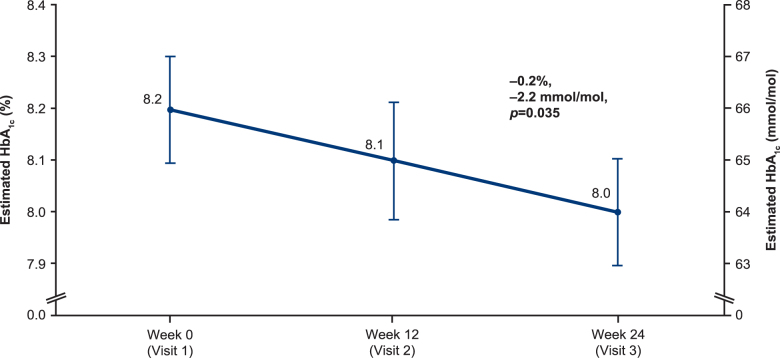
Mean estimated HbA_1c_, EAS-iscCGM (*n* = 92). EAS-iscCGM, effectiveness analysis set patients with sufficient intermittent-scanning continuous glucose monitoring data available; HbA_1c_, glycated hemoglobin.

There were significant reductions from baseline to week 24 in mean PPG (−0.8 mmol/L [−15.1 mg/dL (SD 43.0)], *P* = 0.002), FPPG (−0.8 mmol/L [−13.8 mg/dL (SD 44.0)], *P* = 0.005), and estimated HbA_1c_ (−0.2% [0.8], 2.2 [8.7] mmol/mol, *P* = 0.035) (Table 2, FAS data in Supplementary Table S3). Estimated HbA_1c_ is shown in Figure 2 (EAS) and Supplementary Figure S2 (FAS).

**Table 2.** Aggregated Intermittent-Scanning Continuous Glucose Monitoring Results Over Time, Effectiveness Analysis Set

	iscCGM-EAS^a^
Mean (SD) postprandial glucose, mmol/L [mg/dL]	
*N*^b^	89^b^
Baseline; week 0	11.9 (2.3) [213.7 (41.7)]
Week 12	11.3 (2.3) [204.5 (40.8)]
Week 24	11.0 (2.4) [198.3 (43.9)]
Change in postprandial glucose from week 0 to week 24	–0.8 (2.4) [−15.1 (43.0)], *P* = 0.002
Mean (SD) fasting preprandial glucose (before 9 am), mmol/L [mg/dL]	
*N*^b^	89^b^
Baseline; week 0	8.6 (2.4) [155.3 (43.1)]
Week 12	8.1 (2.3) [146.2 (41.5)]
Week 24	7.8 (2.4) [141.0 (43.0)]
Change in fasting preprandial glucose from week 0 to week 24	–0.8 (2.4) [−13.8 (44.0)], *P* = 0.005
Mean (SD) estimated HbA_1c_, %	
*N*	92
Baseline; week 0	8.2 (1.0)
Week 12	8.1 (1.1)
Week 24	8.0 (1.0)
Change in estimated HbA_1c_ from week 0 to week 24	–0.2 (0.8), *P* = 0.035
Mean (SD) interstitial glucose, mmol/L [mg/dL]	
*N*	92
Baseline; week 0	10.4 (1.6) [187.6 (28.1)]
Week 12	10.3 (1.8) [185.3 (32.2)]
Week 24	10.1 (1.5) [182.2 (27.9)]
Change in mean interstitial glucose from week 0 to week 24	–0.3 (1.3) [−5.4 (24.2)], *P* = 0.035
Mean (SD) coefficient of variation, %	
*N*	92
Baseline; week 0	38.7 (6.6)
Week 12	37.9 (5.8)
Week 24	38.4 (5.6)
Change in mean coefficient of variation from week 0 to week 24	–0.4 (5.7), *P* = 0.541
Mean (SD) MAGE	
*N*	92
Baseline; week 0	162.0 (33.9)
Week 12	155.2 (34.3)
Week 24	154.4 (29.4)
Change in MAGE from week 0 to week 24	–7.5 (32.8), *P* = 0.03

^a^ EAS: included all patients from the SAS without relevant protocol deviations and who continued on treatment until visit 3 of the study.

^b^ *N* numbers for PPG and FPPG were 89 (baseline), 88 (weeks 12 and 24), and 86 (change in value).

mmol/L data calculated by dividing mg/dL data by 18.02.

MAGE, mean amplitude of glycemic excursions.

**FIG. 3.**
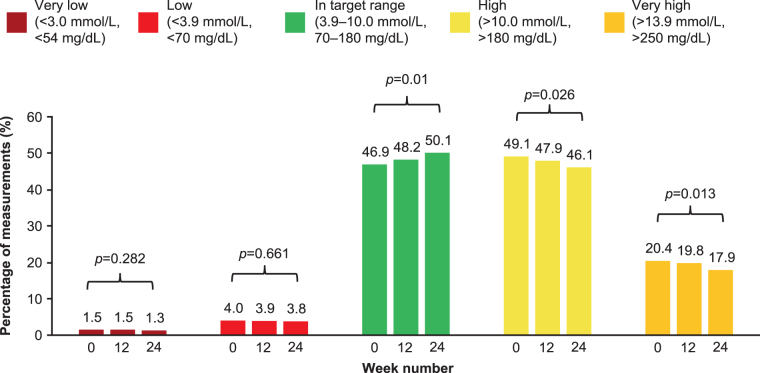
Average percentage of measurements within each glucose range, iscCGM-EAS (*n* = 92). iscCGM-EAS, effectiveness analysis set patients with sufficient intermittent-scanning continuous glucose monitoring data available.

**FIG. 4.**
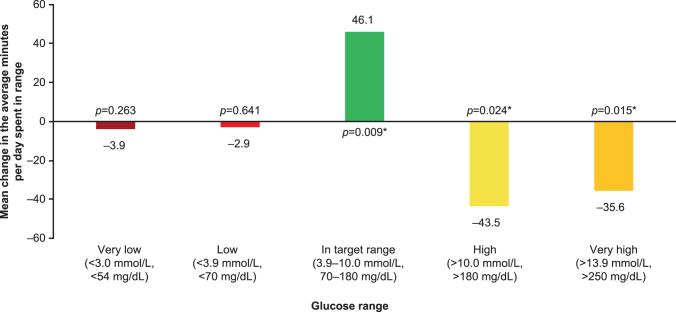
Mean change from baseline to week 24 in average minutes per day spent in each glucose range, iscCGM-EAS (*n* = 92). *Significant difference versus baseline. EAS, effectiveness analysis set.

The percentage of measurements within each glucose range is shown in Figure 3 (FAS data in Supplementary Fig. S3). The changes between baseline to week 24 were as follows: the percentage of measurements in the target glucose range (3.9–10 mmol/L [70–180 mg/dL]) increased from 46.9% to 50.1% (*P* = 0.01), the percentage of measurements that were >10 mmol/L (>180 mg/dL) decreased from 49.1% to 46.1% (*P* = 0.026), while those that were >13.9 mmol/L (>250 mg/dL) decreased from 20.4% to 17.9% (*P* = 0.013) and the percentage of measurements in low or very low glucose ranges remained virtually unchanged. The change in average minutes per day spent in each assessed glucose range is shown in Figure 4 (FAS data in Supplementary Fig. S4). The average minutes per day spent in target range significantly increased by 46.1 min (*P* = 0.009) and was accompanied by a significant decrease in time spent in hyperglycemia (−43.5 min, *P* = 0.024 for >10 mmol/L [>180 mg/dL] threshold and −35.6 min, *P* = 0.015 for >13.9 mmol/L [>250 mg/dL] threshold), while time spent in hypoglycemia remained virtually unchanged compared with baseline.

There were no significant differences in the mean overall number of hypoglycemic episodes, including very low glucose episodes (<3.0 mmol/L [<54 mg/dL]) and low glucose episodes (<3.9 mmol/L [<70 mg/dL]). Overall, the mean interstitial glucose was reduced from baseline at weeks 12 and 24 (Table 2). This reduction was most pronounced at week 24 (−0.3 mmol/L [−5.4 mg/dL; SD 24.2], *P* = 0.035, EAS [Table 2, FAS data in Supplementary Table S3]).

There was only a small mean change in coefficient of variation at weeks 12 and 24 compared with baseline (−0.8 at week 12, −0.4 at week 24; not significant; Table 2). MAGE was significantly reduced at weeks 12 and 24 (−6.7 at week 12 [*P* = 0.03], −7.5 at week 24 [*P* = 0.03]; Table 2).

### Secondary endpoints

#### Fasting plasma glucose

The change in laboratory-measured FPG from baseline to week 24 (−0.2 mmol/L [−3.63 mg/dL; 95% CI: −19.46 to 12.21]) and week 12 (−0.4 mmol/L [−8.10 mg/dL; 95% CI: −22.06 to 5.85]) was small and not statistically significant.

#### Insulin dose

The change from previous therapy to week 24 in basal (0.5 U [95% CI: −0.42 to 1.41]) and bolus (−1.2 U [95% CI: −3.00 to 0.71]) insulin dose was not statistically significant. Similarly, there was no significant difference in the basal/bolus insulin ratio from baseline to week 24 (−8.9 [95% CI −40.36 to 22.65]).

After switching to faster aspart, a small number of patients changed the timing of bolus dosing to after the start of the meal: 3.2% (5/155) at baseline compared with 9.2% (14/153) at week 24.

#### Patient reported outcomes

Quality of life improved from baseline to week 24: the total DTSQ score in the EAS increased by 1.7 (95% CI: 0.71–2.72; *P* = 0.001) and total TRIM-D score by 5.8 (95% CI: 4.08–7.53; *P* < 0.0001) (Table 3 for EAS and FAS).

**Table 3.** Baseline, Week 24, and Change from Baseline to Week 24 Scores for the Diabetes Treatment Satisfaction Questionnaire and the Treatment Related Impact Measure for Diabetes questionnaire, Full Analysis Set, and Effectiveness Analysis Set

	Baseline	Week 24	Change from baseline to week 24	95% CI	P
FAS^a^					
Diabetes treatment satisfaction questionnaire	27.8 (5.6)	29.4 (5.5)	1.7 (6.2)	0.71–2.72	0.001
Treatment-related impact measure for diabetes	69.6 (11.1)	75.4 (11.7)	5.8 (9.7)	4.08–7.53	<0.0001
EAS^b^					
Diabetes treatment satisfaction questionnaire	28.0 (5.6)	29.6 (5.3)	1.7 (6.4)	0.82–2.65	0.001
Treatment-related impact measure for diabetes	69.9 (11.5)	75.5 (11.7)	5.7 (9.9)	4.24–7.25	<0.0001

Data are mean (SD) unless otherwise stated.

^a^ FAS: included all enrolled patients, defined as all those who had signed an informed consent for the study, excluding screening failures.

^b^ EAS: included all patients from the SAS without relevant protocol deviations and who continued on treatment until visit 3 of the study.

CI, confidence interval.

#### Safety data

Overall, five patients reported six SARs until study completion: five SARs in four patients were nonfatal; one was fatal. The fatal event occurred after the patient (54-year-old male) went skiing and paragliding and subsequently experienced very low blood sugar. The cause of death was not listed and it is not possible to ascertain the patient's food intake or insulin dose that day. Of the nonfatal SARs, five (in four patients) were hospitalizations due to ketoacidosis (four events were moderate and one was severe in intensity; three events occurred alongside hyperglycemia of the same intensity as their diabetic ketoacidosis; all the events were assessed as unrelated to the study medication). Altogether, this does not change the current knowledge of the safety profile of faster aspart.

## Discussion

This real-world study in Germany of adults with T1D confirmed findings from the regulatory “onset” trials of improved PPG control with faster aspart leading to statistically significant improvements in HbA_1c_^2,3^ and demonstrated improvements in TIR at 24 weeks without increasing time spent in hypoglycemia. The reduction in HbA_1c_ from baseline in this study is greater than the treatment difference observed between faster aspart and IAsp in the 26- and 52-week data from onset-1 and onset-8.^3,4^ Overall, FAS and EAS data correlated, indicating that these conclusions were not biased by selecting only the most adherent patients. Furthermore, the mean insulin doses and the mean basal/bolus ratio remained constant, supporting the findings from randomized controlled trials that patients could benefit solely from switching bolus insulin.^3,4^ Overall, our findings support the treatment switch to faster aspart in combination with iscCGM in insulin-experienced patients with T1D, particularly in those in need of better glycemic control and/or more flexible bolus administration.

In our study, a full 2-week sensor download with 80% complete data for each visit was available for 92 patients, and, therefore, an aggregated analysis of all single-patient iscCGM metrics could be performed. These metrics provided insights into the glycemic control of patients that would not be apparent from HbA_1c_ evaluations alone.^20^ The iscCGM data included significant reductions in mean interstitial glucose, PPG, and FPPG. Again, this is consistent with the improvements in PPG increment observed with faster aspart in clinical trials after a standardized liquid meal test in adults or in the CGM subgroup of children in onset 7 (both with MDI).^3,11^ The reduction in PPG likely accounts for much of the reduction in time in hyperglycemia, and hence the improved TIR. As the PPG control of patients with T1D is most critically influenced by their bolus insulin dose, these data provide evidence that faster aspart can help to improve overall glycemic control through reduced PPG excursions in a real-world setting.

While HbA_1c_ measurements are routinely included in clinical trials, alone, this metric fails to identify important changes in patients' blood glucose stability or frequent excursions in high or low glucose ranges. TIR is now recognized as an important parameter to measure in patients with diabetes,^15,25–27^ and several studies have demonstrated an association between TIR and the risk of diabetic complications.^28–31^ For example, Beck et al. demonstrated that the hazard rate for retinopathy progression increased by 64% for each 10% reduction in TIR (as calculated from seven-point self-measured blood glucose profiles).^29^ Therefore, the statistically significant improvements in TIR observed with initiation of faster aspart in this study are an important finding. Several parameters have been suggested as a measure of glycemic variability.^20^ While there was no significant difference in coefficient of variation, as an easy-to-calculate parameter, there was a statistically significant difference at week 24 in the MAGE, which is the classic marker of the amplitude of glucose fluctuations. This metric of glycemic variability uses a calculation that is “devoid of time component,” that is to say that it focuses solely on the magnitude of the minimum to maximum glucose levels, regardless of the time it takes to transition from one extreme glucose level to the next.^32^ The impact of glycemic variability on long-term outcomes is controversial, however, some studies using CGM have demonstrated an association between glycemic variability and retinopathy, microalbuminuria, and neuropathy.^33,34^ This in turn highlights the value of reporting data from new technologies, such as iscCGM, to fully understand the effect of an intervention.

It is also important to report that real-world studies, such as GoBolus, look beyond HbA_1c_ and include patient-reported outcomes.^35^ The improved treatment satisfaction observed in the GoBolus DTSQ and TRIM-D analyses may reflect the totality of the improvement in glycemic control, but could also show a key advantage of faster aspart in that it offers increased dosing flexibility compared with other bolus insulins currently available. However, while being statistically significant, changes in DTSQ and TRIM-D were numerically small. As there is no predetermined minimally important change for many patient-reported outcome tools, including these questionnaires, the clinical meaning of these differences remains to be defined.

The key limitations of this study were due to the observational, open-label nature of the trial design, which could have affected study outcomes. Complete and reliable iscCGM data were only available for 92 participants in the EAS (63 had insufficient data quality); a limited sample but reflective of the observational design of our study. Real-world studies are more likely to be subject to bias such as selection bias,^36^ in this instance the decision to prescribe faster aspart may be due to patient or disease characteristics that would be controlled for in a randomized controlled trial. The nature of the study precluded a central laboratory; therefore differences, for example, in local HbA_1c_ measurement procedures cannot be ruled out; however, German national guidelines only allow quality-approved procedures,^37,38^ and therefore the data should be reliable. In addition, as the study did not have a control group, there is no way of confirming how clinical outcomes would be different if the patients had not switched to faster aspart. For example, we cannot exclude that some of the observed improvement in glycemic control was due to patients becoming more adept at using their iscCGM during the course of the study. However, this limitation has been addressed by including only patients who had regularly used their iscCGM device for at least 6 months before study start.

The key strength of the study was the enrolment of patients in a real-world setting, where the decision to initiate faster aspart was made independently of study participation. Inclusion criteria were wide enough to ensure that the study population resembles patients in German clinical practice and possibly countries with similar healthcare systems. Accordingly, the reported outcomes provide important insights into the impact of iscCGM and faster aspart on patients in the clinic, and they merit further study on a global level.

## Conclusion

This study in Germany confirmed the clinical benefits of faster aspart reported in the “‘onset” randomized controlled trials, in a large population of patients with T1D receiving MDI, as well as highlighting the value of iscCGM for illustrating the impact of an intervention. Glycemic control was improved, as demonstrated by both HbA_1c_ and iscCGM data, by reducing glucose excursions and, overall, this was achieved without changing the type of basal insulin or the mean insulin dose.

## Supplementary Material

Supplemental data

Supplemental data

Supplemental data

Supplemental data

Supplemental data

Supplemental data

Supplemental data
