# Supplementary material for: Impact of Fast-Acting Insulin Aspart on Glycemic Control in Patients with Type 1 Diabetes Using Intermittent-Scanning Continuous Glucose Monitoring Within a Real-World Setting: The GoBolus Study
Source: Diabetes Technol Ther. 2021 Feb 25;23(3):203–12. doi: 10.1089/dia.2020.0360 (PMC7906866; doi:10.1089/dia.2020.0360)
Supplement: Supplemental data [file Supp_FigS1.docx]

**Supplementary Figure 1.** Patient disposition


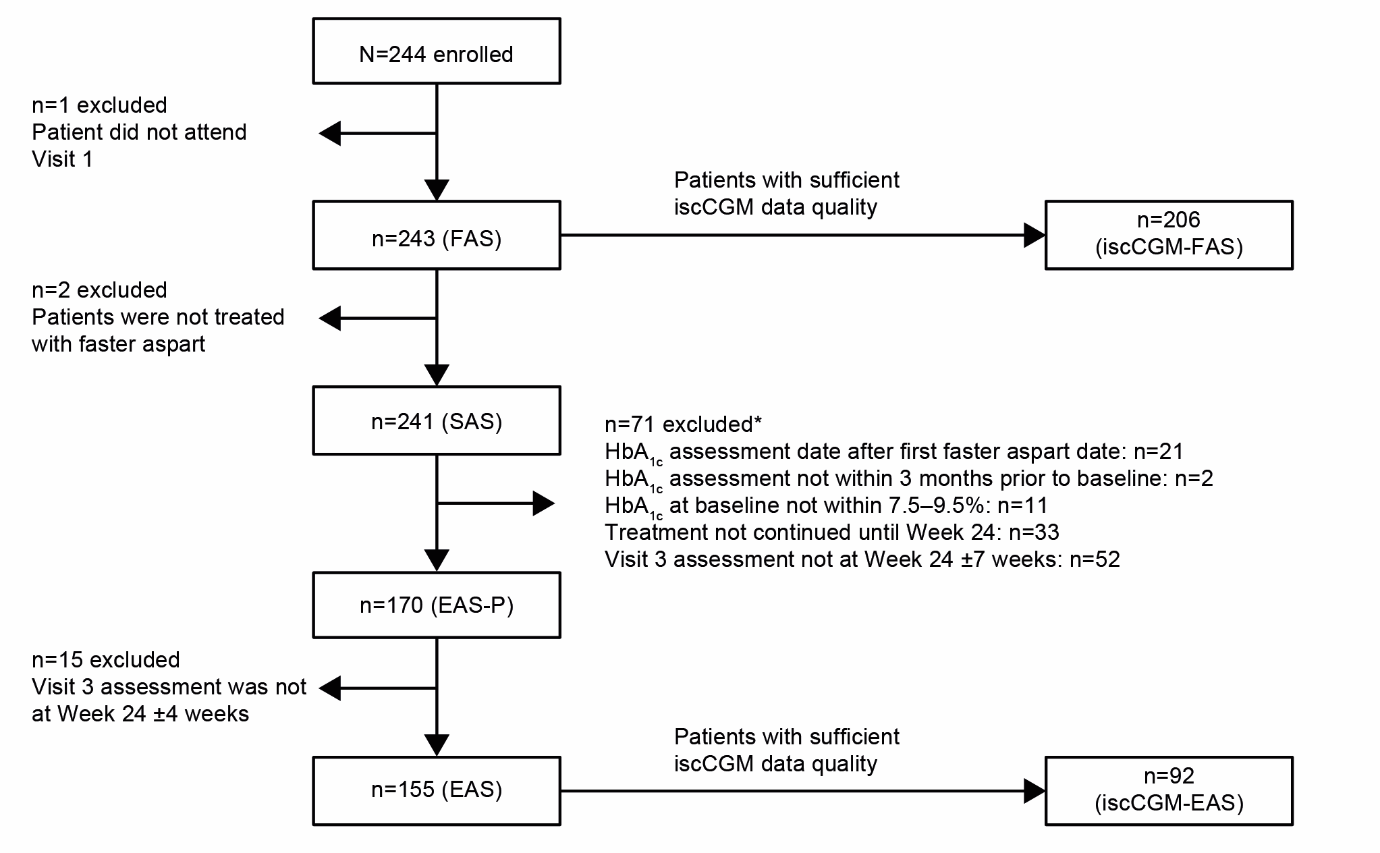


*Some patients had more than one reason for exclusion.

EAS, effectiveness analysis set; EAS-P, extended effectiveness analysis set for the primary endpoint; FAS, full analysis set; iscCGM, intermittent-scanning continuous glucose monitoring; SAS, safety analysis set.
