# Supplementary material for: Impact of Fast-Acting Insulin Aspart on Glycemic Control in Patients with Type 1 Diabetes Using Intermittent-Scanning Continuous Glucose Monitoring Within a Real-World Setting: The GoBolus Study
Source: Diabetes Technol Ther. 2021 Feb 25;23(3):203–12. doi: 10.1089/dia.2020.0360 (PMC7906866; doi:10.1089/dia.2020.0360)
Supplement: Supplemental data [file Supp_TableS1.docx]

**Supplementary Table 1.** Reasons* to initiate the treatment with faster aspart, safety analysis set

| **n (%)** | **Patients**  **(n=241)** |
| --- | --- |
| Improvement of blood glucose profile | 157 (65.1) |
| Insufficient HbA_1c_ improvement | 110 (45.6) |
| Time flexibility in bolus administration | 48 (19.9) |
| Intolerance to previous therapy | 7 (2.9) |
| Other reason for faster aspart initiation | 11 (4.6) |

Safety analysis set: included all patients who had received at least one dose of study treatment.

*More than one reason could be given.

HbA_1c_, glycated hemoglobin.
