# Supplementary material for: Impact of Fast-Acting Insulin Aspart on Glycemic Control in Patients with Type 1 Diabetes Using Intermittent-Scanning Continuous Glucose Monitoring Within a Real-World Setting: The GoBolus Study
Source: Diabetes Technol Ther. 2021 Feb 25;23(3):203–12. doi: 10.1089/dia.2020.0360 (PMC7906866; doi:10.1089/dia.2020.0360)
Supplement: Supplemental data [file Supp_TableS2.docx]

**Supplementary Table 2.** Reasons for premature discontinuation of treatment, safety analysis set

| **n (%)** | **Patients**  **(n=241)** |
| --- | --- |
| Not completed | 40 (16.6) |
| Lost to follow-up | 10 (4.1) |
| Death | 1 (0.4) |
| Withdrew consent | 1 (0.4) |
| Other | 28 (11.6) |

Safety analysis set: included all patients who had received at least one dose of study treatment.
