# Supplementary material for: Impact of Fast-Acting Insulin Aspart on Glycemic Control in Patients with Type 1 Diabetes Using Intermittent-Scanning Continuous Glucose Monitoring Within a Real-World Setting: The GoBolus Study
Source: Diabetes Technol Ther. 2021 Feb 25;23(3):203–12. doi: 10.1089/dia.2020.0360 (PMC7906866; doi:10.1089/dia.2020.0360)
Supplement: Supplemental data [file Supp_TableS3.docx]

**Supplementary Table 3.** Aggregated intermittent-scanning continuous glucose monitoring results for patients receiving multiple daily injections over time, FAS

|  | **isCGM-FAS^a^** |
| --- | --- |
| **Mean (SD) postprandial glucose, mmol/L** | |
|  |  |
| Week 0 (n=199) | 11.5 (2.2) [208.1 (39.8)] |
| Week 12 (n=160) | 11.0 (2.3) [198.8 (41.8)] |
| Week 24 (n=136) | 10.9 (2.3) [197.0 (40.8)] |
| Change in postprandial glucose from Week 0 to Week 24 (n=124) | –0.7 (2.4) [–13.1 (42.8)]  *p*=0.001 |
| **Mean (SD) fasting pre-prandial glucose (before 9 am), mmol/L** | |
|  |  |
| Week 0 (n=199) | 8.3 (2.3) [148.8 (41.2)] |
| Week 12 (n=160) | 7.8 (2.4) [140.0 (42.4)] |
| Week 24 (n=136) | 7.7 (2.3) [138.5 (41.6)] |
| Change in fasting pre-prandial glucose from Week 0 to Week 24 (n=124) | –0.7 (2.4) [–13.8 (44.0)]  *p*=0.003 |
| **Mean (SD) estimated HbA_1c_ , %** | |
|  |  |
| Week 0 (n=206) | 8.1 (1.0) |
| Week 12 (n=165) | 8.0 (1.1) |
| Week 24 (n=143) | 7.9 (1.0) |
| Change in estimated HbA_1c_ from Week 0 to Week 24 (n=132) | –0.2 (0.9)  *p*=0.023 |
| **Mean (SD) interstitial glucose, mmol/L** | |
|  |  |
| Week 0 | 10.2 (1.6) [184.5 (29.4)] |
| Week 12 | 10.2 (1.8) [183.2 (32.9)] |
| Week 24 | 10.0 (1.5) [180.5 (27.6)] |
| Change in mean interstitial glucose from Week 0 to Week 24 | –0.3 (1.4) [–5.0 (25.1)]  *p*=0.023 |
| **Mean (SD) coefficient of variation, %** | |
|  |  |
| Week 0 (n=206) | 39.1 (7.0) |
| Week 12 (n=165) | 38.5 (6.6) |
| Week 24 (n=143) | 38.4 (6.7) |
| Change in mean coefficient of variation from Week 0 to Week 24 (n=132) | –0.3 (5.7)  *p*=0.616 |
| **Mean (SD) MAGE** | |
|  |  |
| Week 0 (n=206) | 160.8 (37.0) |
| Week 12 (n=165) | 155.8 (37.7) |
| Week 24 (n=143) | 153.1 (32.2) |
| Change in MAGE from Week 0 to Week 24 (n=132) | –7.2 (31.2)  *p*=0.009 |

^a^FAS: included all enrolled patients, defined as all those who had signed an informed consent for the study, excluding screening failures.

mmol/L data calculated by dividing mg/dL data by 18.02.

FAS, full analysis set; iscCGM-FAS, patients with intermittent-scanning continuous glucose monitoring data in the full analysis set; MAGE, mean amplitude of glycemic excursions; SD, standard deviation.
