# Supplementary material for: Impact of Fast-Acting Insulin Aspart on Glycemic Control in Patients with Type 1 Diabetes Using Intermittent-Scanning Continuous Glucose Monitoring Within a Real-World Setting: The GoBolus Study
Source: Diabetes Technol Ther. 2021 Feb 25;23(3):203–12. doi: 10.1089/dia.2020.0360 (PMC7906866; doi:10.1089/dia.2020.0360)
Supplement: Supplemental data [file Supp_FigS4.docx]

**Supplementary Figure 4.** Mean change from baseline to Week 24 in average minutes per day spent in each glucose range, iscCGM-FAS (n=132)


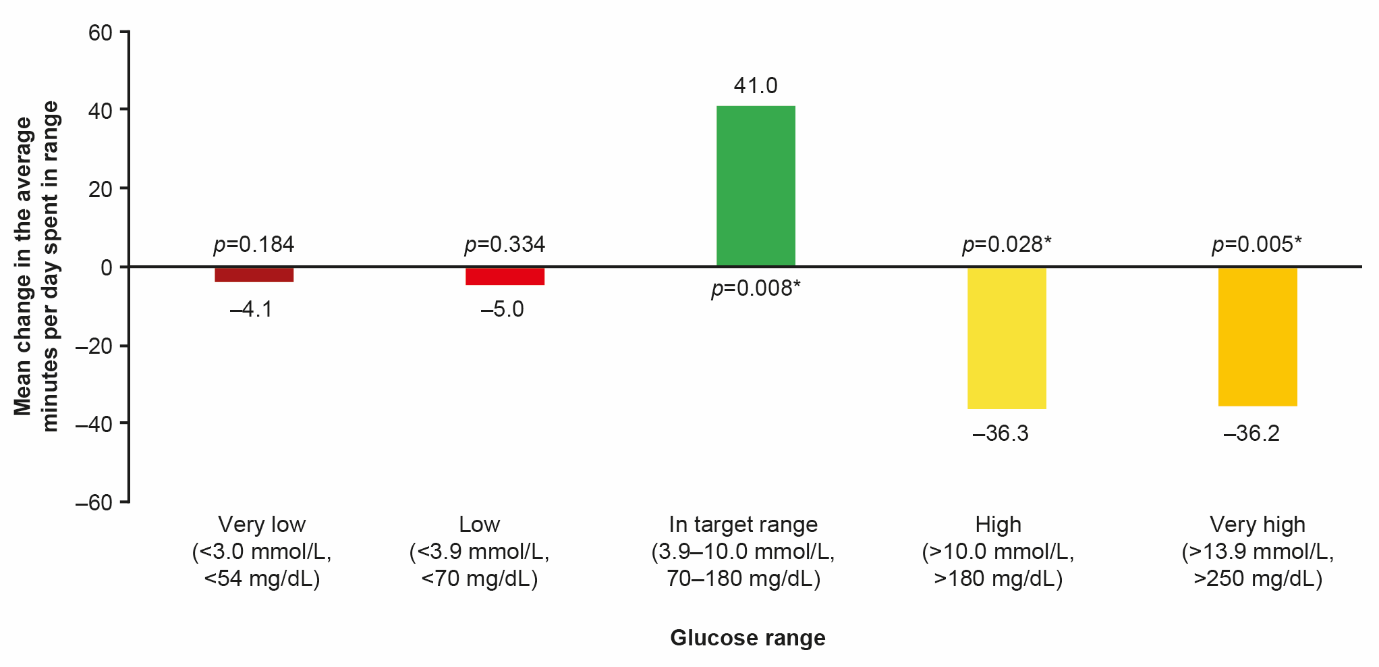


*Significant difference versus baseline

iscCGM-FAS, full analysis set patients with sufficient intermittent-scanning continuous glucose monitoring data available.
