# Supplementary material for: Impact of Fast-Acting Insulin Aspart on Glycemic Control in Patients with Type 1 Diabetes Using Intermittent-Scanning Continuous Glucose Monitoring Within a Real-World Setting: The GoBolus Study
Source: Diabetes Technol Ther. 2021 Feb 25;23(3):203–12. doi: 10.1089/dia.2020.0360 (PMC7906866; doi:10.1089/dia.2020.0360)
Supplement: Supplemental data [file Supp_FigS3.docx]

**Supplementary Figure 3.** Average percentage of measurements within each glucose range, iscCGM-FAS (n=206 [baseline], n=165 [Week 12], n=143 [Week 24])

**
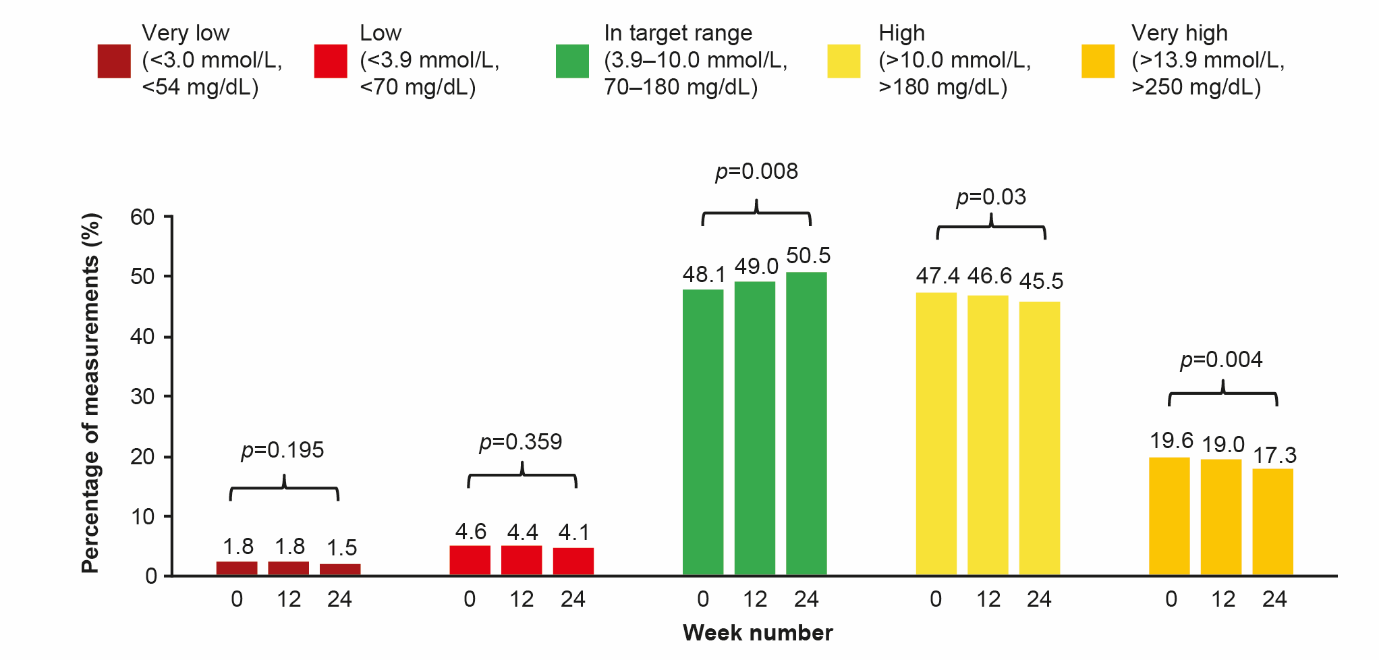
**

iscCGM-FAS, full analysis set patients with sufficient intermittent-scanning continuous glucose monitoring data available.
